# Wide scan imaging with swept-source optical coherent tomography for glaucoma diagnosis

**DOI:** 10.1371/journal.pone.0195040

**Published:** 2018-04-05

**Authors:** Eun Hee Hong, Yong Un Shin, Min Ho Kang, Heeyoon Cho, Mincheol Seong

**Affiliations:** Department of Ophthalmology, Hanyang University College of Medicine, Seoul, Korea; Massachusetts Eye & Ear Infirmary, Harvard Medical School, UNITED STATES

## Abstract

**Purpose:**

To determine glaucoma-discriminating abilities of macular and circumpapillary retinal nerve fiber layer (cpRNFL) thickness measurements of wide scan (12X9mm) using swept-source optical coherence tomography (SS-OCT) compared to measurements of standard macula and disc scans (6 X 6 mm).

**Methods:**

This retrospective chart review study included 60 glaucomatous and 62 healthy eyes of total 122 subjects who visited a glaucoma clinic and were examined with wide, standard macula, and standard disc scans of SS-OCT (DRI-OCT-1 Atlantis; Topcon Inc., Tokyo, Japan) on the same day. Thickness measurements of the ganglion cell layer plus inner plexiform layer (mGCIPL), mGCIPL plus nerve fiber layer (mGCC), and total retinal layer (TRL) were assessed in wide and standard macula scans. Thickness measurements of cpRNFL were assessed in wide and standard disc scans. The repeatability and agreement of measurements taken in each scan were evaluated using intraclass correlation coefficients (ICCs). The abilities of parameters to discriminate between glaucoma and normal groups were assessed using areas under receiver operating characteristic curves (AUCs).

**Results:**

The repeatability and agreement of all parameters showed high ICC values (all ≥ 0.800). AUCs for mGCIPL thickness were 0.710–0.847 and 0.701–0.836 in standard macula and wide scans, respectively. AUCs for cpRNFL thickness were 0.749–0.902 and 0.726–0.897 in standard disc and wide scans, respectively. There were no significant differences in AUCs between wide and standard scans.

**Conclusions:**

The agreement between SS-OCT wide and standard scans for mGCIPL, mGCC and cpRNFL measurements were excellent. As the glaucoma-discriminating ability of wide scans was comparable to that of standard macula/disc scans, a single wide scan can replace separate standard macula/disc scans for evaluating glaucoma.

## Introduction

Measuring structural changes is essential for diagnosing and evaluating glaucoma,[1, 2] and optical coherence tomography (OCT) is a well-established method for objectively assessing the structural changes in eyes with glaucoma.[3, 4] Theoretically, circumpapillary retinal nerve fiber layer (cpRNFL) thickness reflects global RNFL damage and the macular ganglion cell layer (mGCC) thickness reflects the loss of retina ganglion cells (RGCs) in the macular, both of which are useful and complementary markers of glaucomatous damage. In addition to the importance of the cpRNFL, there is a general consensus that measurements of both cpRNFL and mGCC are required, as the importance of mGCC in glaucoma assessment increases.[5] Therefore, in conventional OCT, two scans of the macular and circumpapillary area had to be performed separately to assess mGCC and cpRNFL, respectively.

With advances in OCT technology, the newly-introduced swept-source OCT (SS-OCT) provides higher resolution images, with a center wavelength of 1050 nm and a sweeping range of approximately 100nm, compared to the fixed 850 nm wavelength typical of spectral-domain OCT (SD-OCT).[6, 7] Recently, several studies comparing the diagnostic ability of SS-OCT to SD-OCT in glaucoma showed similar diagnostic ability[8–10] and another study showed good repeatability of SS-OCT.[11] One of the advantages of SS-OCT is the wide scan mode, covering a 12 X 9 mm area of the posterior pole, including the macula and optic nerve head in a single scan. SS-OCT also incorporates automated segmentation software, which makes it possible to image and analyze cpRNFL and the thickness of each macular layer using a single scan. As in conventional SD-OCT, there are also standard macula and disc scan modes in SS-OCT. Previous SS-OCT studies used SS-OCT wide scans[9–11] or SS-OCT standard macula and disc scans[8] to compare thickness measurements and diagnostic abilities in glaucoma of SS-OCT to conventional SD-OCT standard macula or disc scans.

As no previous studies have compared SS-OCT wide scans to SS-OCT standard macula or disc scans, we investigated the glaucoma-discriminating ability of mGCC and cpRNFL thickness measurements taken using SS-OCT wide scans and compared it with that of mGCC thickness measurements using SS-OCT standard macula scans and cpRNFL thickness using SS-OCT standard disc scans.

## Materials and methods

This retrospective cross-sectional study (chart review) included normal subjects and glaucoma patients who visited the Glaucoma Clinic of the Department of Ophthalmology of Hanyang University Guri Hospital between December 2015 and September 2016. The study protocol complied with the Declaration of Helsinki and was approved by the Institutional Review Board (IRB) of Hanyang University Guri Hospital (IRB FILE No. 2016-11-004). The IRB waived the need for consent, and data was accessed anonymously.

### Subjects

After reviewing electronic medical records, normal subjects and glaucoma patients who were examined with both wide and standard macula scans or both wide and standard disc scans of SS-OCT (Topcon DRI OCT-1 Atlantis; Topcon Inc, Tokyo, Japan) twice on the same day were included in this study. We excluded subjects who showed any retinal pathology, neurologic diseases known to affect RNFL thickness or visual fields, high myopic or hyperopic refractive errors of less than –6.0 diopters (D) or greater than +6.0 D, any measurement errors in SS-OCT images, or poor-quality SS-OCT images (DRI SS-OCT image quality score <60).

Eyes were classified as glaucomatous if glaucomatous optic nerve damage (neuroretinal rim notching or thinning, or presence of an RNFL defect) and associated visual field defects were present. A glaucomatous visual field defect was defined when at least one of the following conditions was satisfied: (1) outside normal limits on a glaucoma hemifield test; (2) three contiguous non-edge points (allowing for two nasal-step edge points) with a probability of <5% of being normal and one with a probability of <1% based on pattern deviation; or (3) a pattern standard deviation of <5%. Cases with fixation loss rates > 20% and false-positive and false-negative error rates > 15% were excluded. Healthy subjects were defined as those having intraocular pressure (IOP) of < 21mmHg, a normal-appearing optic disc, and normal visual field results with no history of increased IOP.

### Ocular examinations

All subjects underwent complete ophthalmic examinations. Examinations included a complete medical history assessment and determination of Snellen best-corrected visual acuity, IOP (Goldman applanation tonometry), and refractive spherical equivalent (SE). Slit-lamp biomicroscopy, gonioscopy, and stereoscopic optic disc examination using 90D lens were also performed. Central corneal thickness was measured using ultrasonic pachymetry (Sp-300, Tomey Co., Nagoya, Japan). Visual field (VF) tests using the central 30–2 SITA-Standard strategy with a Humprey field analyzer (model 750, Carl Zeiss Meditec, Dublin, CA, USA), RNFL and optic disc photographs (KOWA VX-10, Kowa Company Ltd., Tokyo, Japan), and SS-OCT imaging were performed.

### SS-OCT protocol

All OCT examinations were performed by a single experienced technician. Pupil dilation was induced in patients before examination. The SS-OCT wide scan covers a 12 x 9 mm area of the posterior pole, which comprises 256 B-scans, each comprising 512 A scans (512 X 256 A-scans, 512 A-scans for each of 256 B-scans) for a total of 131,072 axial scans/volume. In this protocol, 100,000 A-scans are acquired per second for an axial resolution of 8 μm and lateral resolution of 20 μm for the wide-angle scan.[6] The SS-OCT standard macula scan (horizontal cube scan) covers an area of 7 X 7 mm centered on the fovea with 512 X 256 A-scans. The SS-OCT standard disc scan (horizontal disc circle grid scan) covers an area of 6 X 6 mm focused on the optic disc, and cpRNFL thickness was then measured by automated placement of a circle 3.4 mm in diameter, centered on the disc. Manual adjustments of circle placement were performed if necessary.

**Fig 1:**
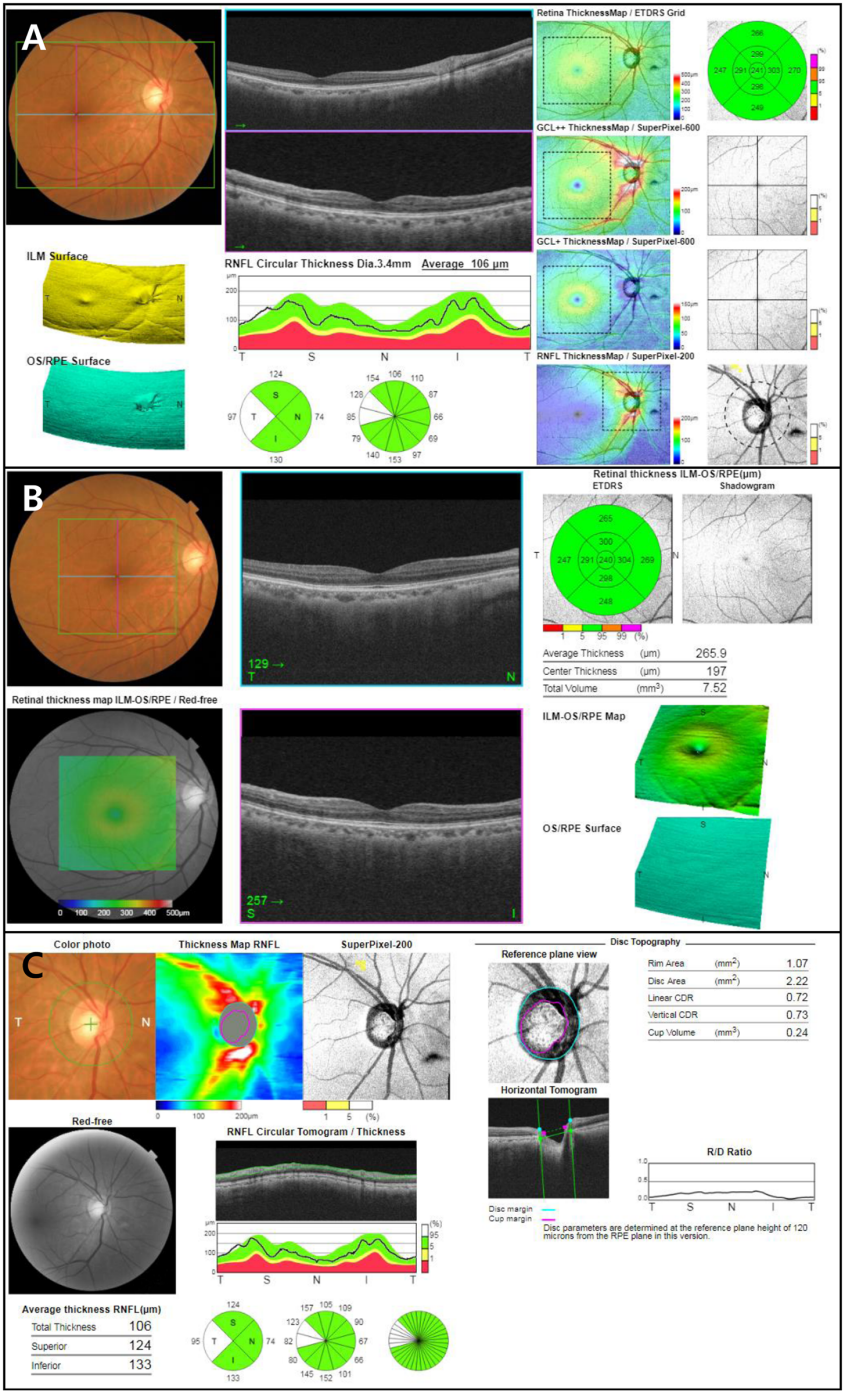
Examples of Topcon SS-OCT reports for each scan in a healthy eye. (A) Wide scan report, (B) standard macula scan report and (C) standard disc scan report. Wide report provides both macular and circumpapillary parameters.

For the analysis of macula area, the thickness of the ganglion cell layer plus inner plexiform layer (mGCIPL), mGCIPL plus the nerve fiber layer (mGCC), and total retinal layer (TRL) in the 6 X 6 mm area centered on the fovea were measured by the segmentation algorithm of DRI SS-OCT both in the wide and standard macular scans. We used the average thicknesses of total (6 mm diameter area) and eight out of nine Early Treatment Diabetic Retinopathy Study (ETDRS) subfields (except for central fovea): inner superior (IS), inner nasal (IN), inner inferior (II), inner temporal (IT), outer superior (OS), outer nasal (ON), outer inferior (OI), and outer temporal (OT) composed of the inner, intermediate, and outer rings with diameters of 1, 3, and 6 mm, respectively (Fig 1). For the analysis of circumpapillary area, cpRNFL thickness was measured by automated placement of a circle 3.4 mm in diameter, centered on the disc, both in the wide scan and standard macular scans. We used the average thicknesses of global cpRNFL and superior, inferior, nasal, and temporal quadrants determined using the same segmentation software (Fig 1). Data were exported using the manufacturer’s OCT-Batch utility (version 4.3.0.118).

As measurement errors including segmentation errors or artifacts might affect thickness measurements,[12] all of the images were reviewed by two masked observers (M. S and E.H.H). We excluded all detected measurement errors, even those that appeared in only a single B-scan image. If any images with centration error, fixation error, or segmentation error were detected, that patient was excluded from the sample. Only images that were considered adequate by both masked observers were included in the analysis.

### Statistical analysis

Statistical analyses were conducted using SPSS (version 12 for Windows; SPSS Inc., Chicago, IL, USA). Independent t-tests were used to compare thickness parameters between normal subjects and glaucoma patients. Intraclass correlation coefficients (ICC) were used to determine the repeatability of each scan. The agreements of mGCIPL, mGCC and TRL thickness measurements in total and eight ETDRS subfields between wide and standard macula scans were analyzed using ICC, and the agreement of cpRNFL thickness measurements in the total area and quadrant sectors between wide scans and standard disc scans were also analyzed using ICC. To interpret the degree of agreement, we followed guidelines described by Fleiss and Cohen: an ICC of 0.75–1.00 represents excellent agreement, 0.6–0.75, good agreement, 0.4–0.6, fair agreement, and < 0.4, poor agreement.[13] Bland-Altman graphs were used to graphically represent the differences between thickness measurements obtained with the two scan modes (wide mode and standard macula mode for macula thickness parameters, wide mode and disc mode for cpRNFL thickness). The abilities of these parameters to discriminate between glaucoma and control groups were assessed using areas under the receiver operating characteristic curves (AUCs). For all tests, the level of statistical significance was set at *P* < 0.05.

## Results

Of all subjects who were examined using both wide and standard macula scans or both wide and standard disc scans during the study period (a total of 300 eyes), we excluded 178 eyes because they showed retinal pathology, SE of less than –6.0 D or greater than +6.0 D, measurement errors in SS-OCT images, or poor-quality SS-OCT images. A total of 122 eyes of 122 subjects were included in this study; 60 eyes were glaucomatous and 62 were healthy. All 122 eyes were examined using both wide scan and standard macula scan. All 60 glaucomatous eyes and 48 eyes among 62 healthy eyes were examined using standard disc scan. The demographics and ocular data of each group are summarized in Table 1. The mean age of the total sample was 56.60 ± 13.73 years and there were no differences in age, gender, and IOP between the normal and glaucoma groups. The average (total) thicknesses of mGCIPL, mGCC, and TRL for each group as measured by wide and standard macula scans, and the thickness parameters of cpRNFL for each group as measured by wide and standard disc scans, are also shown in Table 1. Average mGCIPL thickness was 72.40 ± 5.86 μm versus 64.16 ± 7.85 μm in normal and glaucomatous eyes, respectively, by wide scan (*P* < 0.001), and 71.65 ± 5.63 μm versus 64.34 ± 6.66 μm, respectively, by standard macula scan (*P* < 0.001). Total cpRNFL thickness was 109.39 ± 9.33 μm versus 86.44 ± 17.06 μm in normal and glaucomatous eyes, respectively, by wide scan (*P* < 0.001), and 109.49±9.46 μm versus 86.48 ± 15.68μm, respectively, by standard disc scan (*P* < 0.001). The macular thickness and cpRNFL thickness were significantly thinner in glaucoma eyes compared to normal eyes (*P* < 0.001, for all).

**Table 1:** Demographics and ocular data including macular ganglion cell layer plus inner plexiform layer (mGCIPL), mGCIPL plus nerve fiber layer (mGCC) and total retinal layer (TRL) measurements by swept source optical coherence tomography (SS-OCT) wide scan and standard macula scan, and circumpapillary retinal nerve fiber layer (cpRNFL) measurements by SS-OCT wide scan and standard disc scan of normal subjects and glaucoma patients.

	Total		Normal	Glaucoma	*P* value
**N**	122		62	60	
Age (years)*	56.60 ± 13.73		55.42 ± 14.64	57.80 ± 12.73	0.550
**Gender (F : M)**†	64 : 58		35 : 27	29 : 31	0.806
IOP (mmHg)*	14.84±3.11		15.53 ± 2.84	14.12 ± 3.23	0.470
**MD (dB)** *	-3.61 ± 5.42		0.00 ± 0.67	-5.26 ± 5.83	<0.001
**PSD (dB)** *	3.48 ± 2.86		1.90 ± 0.60	4.25 ± 3.17	<0.001
**Macular thickness (μm)**					
Wide scan*	**mGCIPL (Total)**		72.40 ± 5.86	64.16 ± 7.85	<0.001
	**mGCC (Total)**		109.80 ± 8.04	97.35 ± 12.27	<0.001
	**TRL (Total)**		278.17 ± 15.86	267.58 ± 15.77	<0.001
Standard macula scan*	**mGCIPL (Total)**		71.65 ± 5.63	64.34 ± 6.66	<0.001
	**mGCC (Total)**		107.81 ± 8.06	95.56 ± 11.89	<0.001
	**TRL (Total)**		278.18 ± 15.96	267.48 ± 15.77	<0.001
**cpRNFL thickness (μm)**					
Wide scan*	**cpRNFL**	**Total**	109.39 ± 9.33	86.44 ± 17.06	<0.001
		**Temp**	82.61 ± 12.87	72.15 ± 14.65	<0.001
		**Sup**	132.14 ± 14.32	102.05 ± 25.04	<0.001
		**Nas**	82.87±12.58	69.17 ± 16.10	<0.001
		**Inf**	140.43 ± 17.62	102.39 ± 27.65	<0.001
Standard disc scan*	**cpRNFL**	**Total**	109.49 ± 9.46	86.48 ± 15.68	<0.001
		**Temp**	81.90 ± 12.34	70.08 ± 13.69	<0.001
		**Sup**	134.23 ± 14.48	103.98 ± 23.84	<0.001
		**Nas**	82.26±12.41	69.04 ± 14.45	<0.001
		**Inf**	141.23 ± 16.97	102.88 ± 25.92	<0.001

Values are presented as weighted mean ± standard deviation (SD),

*Independent t-test;

^†^Chi-square test. Abbreviations: IOP = intraocular pressure; MD = Mean deviation; PSD = pattern standard deviation; dB = decibels; mGCIPL = ganglion cell layer plus inner plexiform layer; mGCC = mGCIPL plus nerve fiber layer; TRL = total retinal layer; cpRNFL = circumpapillary retinal nerve fiber layer; Temp = Temporal; Sup = Superior; Nas = Nasal; Inf = Inferior

All ICC values were > 0.9 in each repeated scan mode measurement, separately measured in total, normal and glaucoma groups (0.991–0.999 in macular thickness parameters; 0.993–0.998 in cpRNFL thickness parameters, Table 2).

**Table 2:** Repeatability of wide and standard macula scan measurements of macular thickness, and wide and standard disc scans for circumpapillary retinal nerve fiber layer (cpRNFL) thickness parameters.

		**Wide scan**			**Standard macula scan**		
		**Total**	**Normal**	**Glaucoma**	**Total**	**Normal**	**Glaucoma**
**mGCIPL**	**Total**	0.996	0.993	0.996	0.995	0.994	0.996
	**IT**	0.995	0.993	0.997	0.994	0.991	0.993
	**IS**	0.994	0.995	0.995	0.999	0.998	0.999
	**IN**	0.994	0.996	0.995	0.998	0.997	0.998
	**II**	0.996	0.995	0.997	0.998	0.998	0.998
	**OT**	0.995	0.993	0.996	0.995	0.994	0.992
	**OS**	0.991	0.994	0.996	0.997	0.997	0.995
	**ON**	0.994	0.995	0.995	0.997	0.997	0.996
	**OI**	0.994	0.993	0.995	0.996	0.995	0.994
**mGCC**	**Total**	0.996	0.991	0.996	0.992	0.993	0.994
	**IT**	0.997	0.992	0.997	0.999	0.997	0.999
	**IS**	0.995	0.998	0.995	0.998	0.997	0.998
	**IN**	0.996	0.994	0.997	0.998	0.997	0.995
	**II**	0.995	0.995	0.996	0.999	0.999	0.999
	**OT**	0.994	0.989	0.998	0.998	0.996	0.998
	**OS**	0.994	0.996	0.997	0.998	0.997	0.998
	**ON**	0.996	0.992	0.995	0.997	0.990	0.999
	**OI**	0.996	0.994	0.997	0.999	0.995	0.999
**TRL**	**Total**	0.995	0.993	0.994	0.996	0.997	0.992
	**IT**	0.995	0.993	0.996	0.999	0.999	0.998
	**IS**	0.996	0.995	0.996	0.999	0.999	0.998
	**IN**	0.996	0.995	0.996	0.999	0.998	0.998
	**II**	0.997	0.997	0.996	0.999	0.999	0.999
	**OT**	0.995	0.994	0.995	0.999	0.999	0.998
	**OS**	0.996	0.996	0.995	0.998	0.998	0.997
	**ON**	0.996	0.996	0.996	0.999	0.999	0.999
	**OI**	0.996	0.994	0.997	0.998	0.998	0.998
		**Wide scan**			**Standard disc scan**		
		**Total**	**Normal**	**Glaucoma**	**Total**	**Normal**	**Glaucoma**
**cpRNFL**	**Total**	0.997	0.993	0.997	0.997	0.994	0.997
	**Temp**	0.997	0.996	0.998	0.997	0.996	0.998
	**Sup**	0.998	0.995	0.998	0.998	0.996	0.998
	**Nas**	0.997	0.995	0.998	0.998	0.997	0.997
	**Inf**	0.998	0.997	0.998	0.998	0.996	0.998

Abbreviations: mNFL = macular retinal nerve fiber layer; mGCIPL = ganglion cell layer plus inner plexiform layer; mGCC = mGCIPL plus nerve fiber layer; TRL = total retinal layer; cpRNFL = circumpapillary retinal nerve fiber layer; CF = central fovea; IT = inner inferior; IS = inner superior; IN = inner nasal; II = inner inferior; OT = outer temporal; OS = outer superior; ON = outer nasal; OI = outer inferior; Temp = Temporal; Sup = Superior; Nas = Nasal; Inf = Inferior

**Fig 2:**
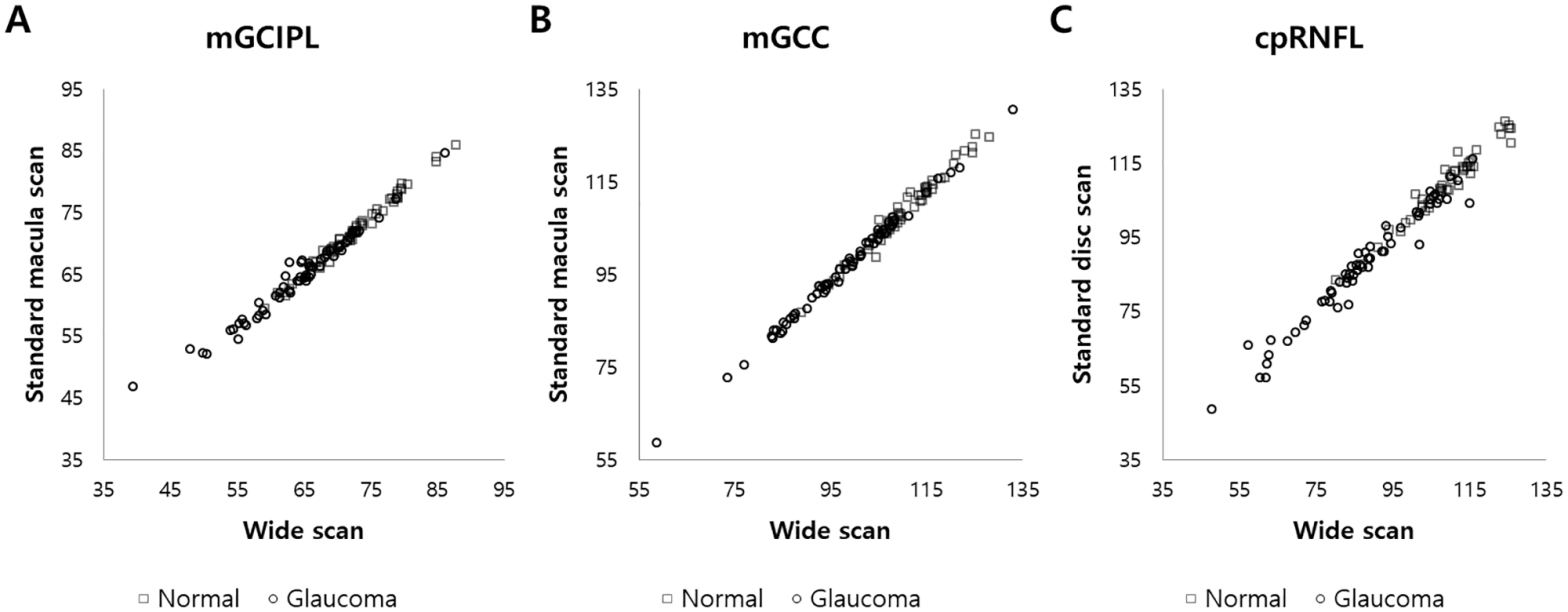
Dot plot graphs with the values from the wide and standard macular/disc scan measurements. Total (average) thickness of (A) ganglion cell layer plus inner plexiform layer (mGCIPL) and (B) macular ganglion cell layer (mGCC), measured by wide scan (X axis) and standard macula scan (Y axis), and total (average) thickness of (C) circumpapillary retinal nerve fiber layer (cpRNFL), measured by wide scan (X axis) and standard disc scan (Y axis), in normal and glaucoma groups are shown.

**Fig 3:**
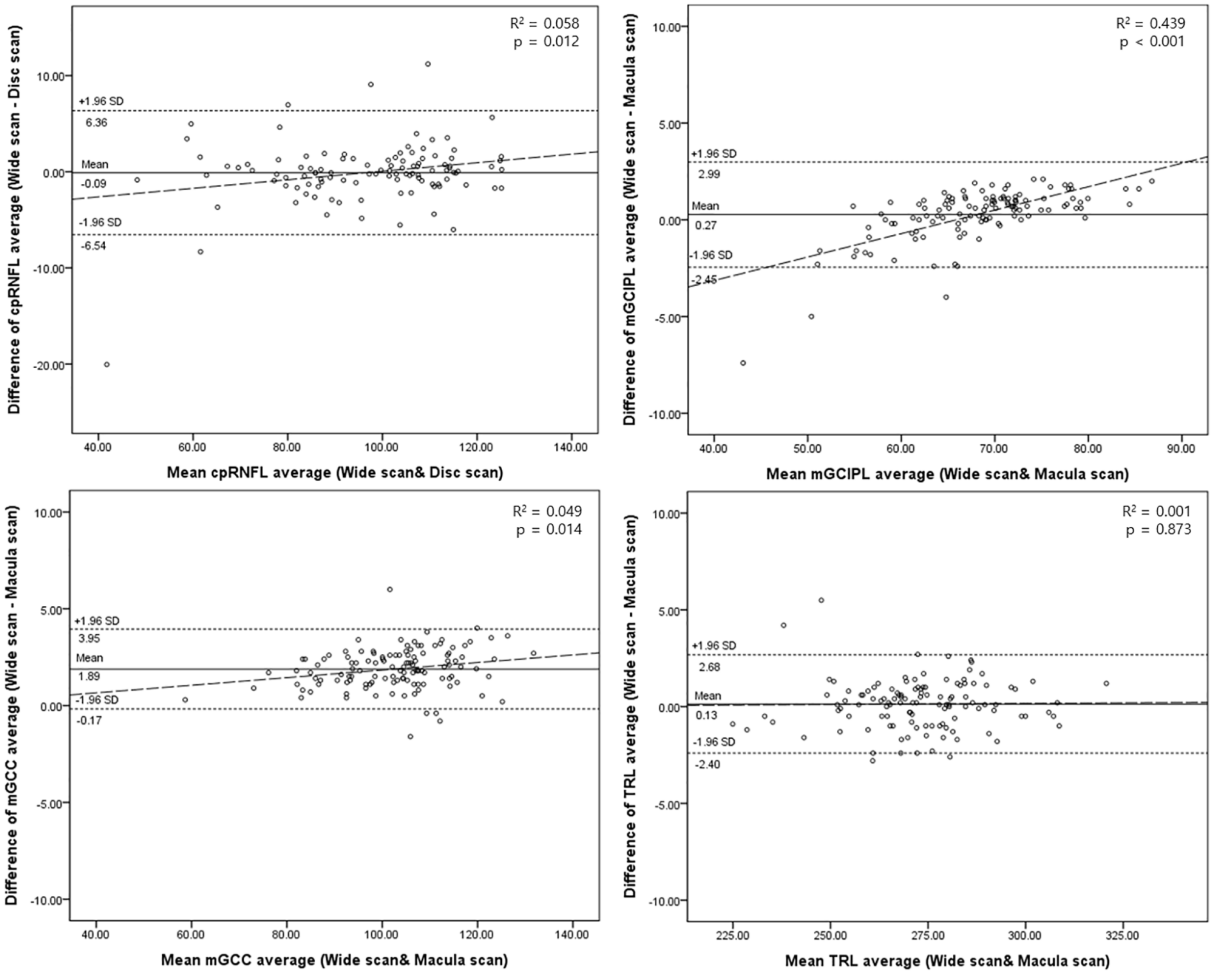
Bland-Altman plots of each macular layer thickness (mGCIPL, mGCC and TRL) and cpRNFL thickness. The plots also show significant correlations in cpRNFL, mGCIPL and mGCC thickness that the difference (Wide–Standard macula or disc) is larger as the measurement value becomes thicker.

The thickness of the total and each ETDRS subfield in each macular layer measured by wide and standard macula scans, and the total and sectoral cpRNFL thickness measured by wide and standard disc scans, are shown in Table 3. The ICC values between wide and standard macula scans for macular thickness parameters, and between wide and standard disc scans for cpRNFL thickness parameters are also shown. For mGCIPL thickness, the ICC values for total and all subfields showed excellent agreement (total, 0.837–0.994; normal, 0.897–0.992; glaucoma 0.800–0.993). For mGCC thickness, the ICC values for total and all subfields also showed excellent agreement (total 0.984–0.995; normal 0.960–0.991; glaucoma 0.981–0.997). For TRL thickness, the ICC values for total and all subfields in all groups showed excellent agreement (0.911–0.997). The ICC values for total and sectoral cpRNFL thickness showed excellent agreement (total, 0.864–0.992; normal, 0.832–0.988; glaucoma, 0.872–0.985). Fig 2 shows wide and standard macula/disc scan measurements of total (average) mGCIPL, mGCC and cpRNFL thickness in normal and glaucoma groups. Fig 3 shows the Bland-Altman plots of thickness for each macular layer (mGCIPL, mGCC and TRL) and cpRNFL thickness. The plots of cpRNFL, mGCIPL, and mGCC thickness also show significant correlation, in that the difference (wide–standard macula or disc scan) becomes larger as the measurement value increases.

**Table 3:** Agreement between wide and standard macula scan measurements for macular thickness parameters, and wide and standard disc scan for circumpapillary retinal nerve fiber layer (cpRNFL) thickness parameters.

	**Total (n = 122)**			**Normal (n = 62)**			**Glaucoma (n = 60)**		
	**Wide**	**Macula**	**ICC**	**Wide**	**Macula**	**ICC**	**Wide**	**Macula**	**ICC**
**mGCIPL**									
**Tot**	68.35±8.03	67.99±7.15	0.983	72.40±5.86	71.65±5.63	0.985	64.16±7.85	64.34±6.66	0.972
**IT**	82.90±12.01	85.80±10.65	0.938	88.84±8.00	91.15±6.86	0.897	76.77±12.42	80.27±11.08	0.928
**IS**	87.92±11.35	87.92±10.92	0.955	91.98±9.22	91.97±8.66	0.992	83.72±11.88	83.74±11.49	0.927
**IN**	87.36±11.67	88.42±10.75	0.983	90.65±9.75	91.70±9.53	0.981	83.97±11.99	85.03±10.96	0.982
**II**	86.27±12.92	85.93±12.77	0.994	91.65±9.34	91.55±8.95	0.991	80.72±13.80	80.11±13.57	0.993
**OT**	64.71±9.52	67.04±7.28	0.889	69.77±6.08	70.80±6.86	0.954	59.48±9.64	63.16±6.74	0.800
**OS**	62.18±7.53	61.74±6.95	0.837	65.45±6.68	64.93±6.28	0.991	**58.80±6.88**	**58.43±6.03**	**0.925**
**ON**	68.43±8.23	68.30±7.78	0.983	71.44±6.85	71.28±6.63	0.991	65.32±8.43	65.22±7.73	0.972
**OI**	56.92±7.49	56.49±7.04	0.960	60.90±53.43	60.32±5.43	0.976	52.80±7.12	52.54±6.51	0.920
**mGCC**									
**Tot**	103.67±12.05	101.79±11.81	0.984	109.80±8.04	107.81±8.06	0.960	97.35±12.27	95.56±11.89	0.987
**IT**	105.14±13.44	104.81±13.34	0.994	111.81±9.16	111.52±8.90	0.985	98.25±13.75	97.78±13.64	0.995
**IS**	116.17±13.97	116.75±13.76	0.992	**121.05±11.65**	**121.69±11.40**	**0.987**	111.13±14.47	111.56±14.20	0.993
**IN**	112.84±13.11	112.29±13.39	0.994	116.65±11.69	116.19±12.04	0.990	108.90±13.43	108.20±13.61	0.997
**II**	115.11±16.12	115.14±16.17	0.995	112.82±12.03	112.13±11.80	0.989	108.18±16.94	107.80±16.94	0.996
**OT**	88.19±11.68	87.14±11.28	0.993	94.71±7.03	93.43±7.04	0.980	81.45±11.64	80.53±11.15	0.995
**OS**	101.77±12.35	101.73±12.40	0.993	107.32±9.16	107.49±9.31	0.987	96.03±12.65	95.68±12.42	0.994
**ON**	117.71±13.86	117.64±13.86	0.986	122.89±10.19	122.95±10.49	0.991	112.37±15.15	112.06±14.82	0.981
**OI**	96.57±15.05	96.40±15.30	0.995	104.53±8.52	104.63±8.65	0.985	88.35±15.93	87.75±16.03	0.996
**TRL**									
**Tot**	272.78±16.64	272.64±16.62	0.997	278.17±15.86	278.18±15.96	0.997	267.48±15.77	267.20±15.53	0.996
**IT**	296.98±19.37	297.14±19.37	0.987	303.22±17.34	303.22±17.28	0.986	290.54±19.37	290.87±19.54	0.986
**IS**	308.18±20.40	309.75±19.96	0.993	312.58±19.87	313.94±19.50	0.994	303.63±20.11	305.42±19.66	0.991
**IN**	309.30±19.44	310.26±19.80	0.990	312.64±19.22	313.18±19.69	0.994	306.46±19.33	307.25±19.62	0.985
**II**	304.10±21.47	305.44±20.50	0.956	309.41±21.21	311.57±18.48	0.911	298.61±20.50	299.09±20.69	0.993
**OT**	253.85±16.61	251.74±16.48	0.964	259.62±15.59	257.16±16.28	0.935	247.88±15.60	246.15±14.82	0.988
**OS**	267.04±17.31	267.36±17.07	0.988	271.76±17.74	272.03±17.78	0.987	262.16±15.54	262.52±14.96	0.988
**ON**	284.21±19.17	284.47±19.08	0.986	287.80±18.59	288.62±18.07	0.975	280.50±19.21	280.19±19.29	0.995
**OI**	252.75±17.14	252.14±17.23	0.988	259.55±14.70	259.21±15.33	0.988	245.73±16.73	244.84±16.09	0.989
	**Total (n = 108)**			**Normal (n = 48)**			**Glaucoma (n = 60)**		
	**Wide**	**Disc**	**ICC**	**Wide**	**Disc**	**ICC**	**Wide**	**Disc**	**ICC**
**cpRNFL**									
**Total**	98.10±17.85	96.71±17.51	0.992	109.39±9.33	109.49±9.46	0.988	86.44±17.06	86.48±15.68	0.985
**Temp**	77.46±14.69	75.33±14.32	0.917	82.61±12.87	81.90±12.34	0.832	72.15±14.65	70.08±13.69	0.949
**Sup**	117.34±25.25	117.42±25.17	0.983	132.14±14.32	134.23±14.48	0.941	102.05±25.04	103.98±23.84	0.983
**Nas**	75.27±16.09	74.08±15.03	0.864	**82.87±12.58**	**82.26±12.41**	**0.952**	69.17±16.10	69.04±14.45	0.872
**Inf**	121.82±29.90	119.93±29.39	0.983	140.43±17.62	141.23±16.97	0.966	102.39±27.65	102.88±25.92	0.973

Abbreviations: mNFL = macular retinal nerve fiber layer; mGCIPL = ganglion cell layer plus inner plexiform layer; mGCC = mGCIPL plus nerve fiber layer; TRL = total retinal layer; cpRNFL = circumpapillary retinal nerve fiber layer; CF = central fovea; IT = inner inferior; IS = inner superior; IN = inner nasal; II = inner inferior; OT = outer temporal; OS = outer superior; ON = outer nasal; OI = outer inferior; Temp = Temporal; Sup = Superior; Nas = Nasal; Inf = Inferior

AUCs showing the ability of each scan mode to distinguish eyes with and without glaucoma are presented in Table 4. Overall, the best parameter was total cpRNFL thickness using standard disc scans (AUC = 0.902). Among average thickness parameters, the cpRNFL thickness was the best in both wide and standard disc scans (AUC = 0.886 and 0.902, respectively), followed by mGCC thickness in both wide and standard macula scans (AUC = 0.822 and 0.827, respectively), and mGCIPL thickness (AUC = 0.815 and 0.815, respectively). For mGCIPL and mGCC thickness, the best two parameters in both wide and standard macula scan were OT and OI (mGCIPL wide, 0.836 and 0.824, respectively; mGCIPL standard macula, 0.836 and 0.847, respectively; mGCC wide, 0.854 and 0.828, respectively; mGCC standard macula, 0.848 and 0.830, respectively). In cpRNFL thickness, the best two parameters in both wide and standard disc scans were total thickness and inferior quadrant thickness (wide, 0.886 and 0.897, respectively; disc, 0.902 and 0.898, respectively). The worst two parameters in mGCIPL in both wide and standard macula scans were IN and ON (wide, 0.701 and 0.702, respectively; standard macula, 0.710 and 0.716, respectively). The worst two parameters in mGCC, in both wide and standard macula scans, were IS and IN (wide, 0.724 and 0.710, respectively; standard macula, 0.733 and 0.702, respectively). The worst parameter in cpRNFL in both wide and standard disc scans was temporal quadrant thickness (wide, 0.726; standard disc, 0.749). There were no significant differences in ability to diagnose glaucoma between wide and standard macula/disc scans in all measurements.

**Table 4:** Comparison of areas under the receiver operating characteristic curves (AUCs) between wide and standard macula scans of macular thickness parameters and between wide and standard disc scans of circumpapillary retinal nerve fiber layer (cpRNFL) thickness parameters for assessing glaucoma.

		**Wide scan**	**Standard macula scan**	***P* value**
**mGCIPL**	**Total**	0.815	0.815	0.999
	**IT**	0.805	0.818	0.342
	**IS**	0.729	0.738	0.262
	**IN**	0.701	0.710	0.262
	**II**	0.763	0.769	0.319
	**OT**	**0.836**	**0.836**	0.962
	**OS**	0.755	0.767	0.226
	**ON**	0.702	0.716	0.091
	**OI**	**0.824**	**0.847**	0.087
**mGCC**	**Total**	0.822	0.827	0.332
	**IT**	0.809	0.806	0.752
	**IS**	0.724	0.733	0.363
	**IN**	0.710	0.702	0.272
	**II**	0.763	0.768	0.544
	**OT**	**0.854**	**0.848**	0.364
	**OS**	0.759	0.775	0.063
	**ON**	0.735	0.739	0.504
	**OI**	**0.828**	**0.830**	0.779
**TRL**	**Total**	0.706	0.696	0.089
	**IT**	0.700	0.694	0.428
	**IS**	0.622	0.621	0.857
	**IN**	0.602	0.594	0.384
	**II**	0.672	0.684	0.376
	**OT**	0.708	0.694	0.377
	**OS**	0.645	0.645	0.981
	**ON**	0.613	0.627	0.110
	**OI**	0.743	0.748	0.588
		**Wide scan**	**Standard disc scan**	***P* value**
**cpRNFL**	**Total**	**0.886**	**0.902**	0.054
	**Temp**	0.726	0.749	0.157
	**Sup**	0.847	0.865	0.240
	**Nas**	0.776	0.760	0.624
	**Inf**	**0.897**	**0.898**	0.241

Abbreviations: mNFL = macular retinal nerve fiber layer; mGCIPL = ganglion cell layer plus inner plexiform layer; mGCC = mGCIPL plus nerve fiber layer; TRL = total retinal layer; cpRNFL = circumpapillary retinal nerve fiber layer; CF = central fovea; IT = inner inferior; IS = inner superior; IN = inner nasal; II = inner inferior; OT = outer temporal; OS = outer superior; ON = outer nasal; OI = outer inferior; Temp = Temporal; Sup = Superior; Nas = Nasal; Inf = Inferior

## Discussion

In this study, we assessed the agreement between wide and standard macula scans used for measuring the thickness of the macular ganglion cell layer, and between wide and standard disc scans for measuring cpRNFL thickness. We also compared the glaucoma-discriminating abilities of parameters measured using wide scan versus standard macula/disc scan. Our results indicate that wide scans show good agreement with standard macula/disc scans, and that the diagnostic ability of wide scans is comparable to that of standard macula/disc scans for assessing glaucoma.

Before the introduction of wide scan SS-OCT, standard macula and disc scans, which must be performed separately, were both needed to evaluate glaucoma patients and to assess mGCC and cpRNFL thickness, respectively. A previous study assessed the diagnostic ability of wide imaging with SD-OCT in glaucoma,[14] but the wide area used (8 mm diameter) did not include the circumpapillary area. The wide scan (12 X 9 mm) mode of SS-OCT covers both the macular and circumpapillary areas, and the incorporated automated segmentation software makes it possible to analyze the cpRNFL and mGCC thicknesses simultaneously using data from a single wide scan. Thus, a single wide scan may replace separate standard macula and disc scans, if the diagnostic power and accuracy of wide scan is comparable. There have been a few studies evaluating the agreement of SS-OCT wide scan measurements and SD-OCT measurements,[11] and comparing the diagnostic ability of SS-OCT wide scan parameters and SD-OCT parameters.[9, 10] Lee et al. used SS-OCT wide scan and SD-OCT standard macula scan to measure mGCC thickness and standard disc scan of SS-OCT and SD-OCT to measure cpRNFL thickness. They then evaluated intradevice repeatability and interdevice agreement of thickness measurements, but only in healthy subjects. Their results showed that SS-OCT had excellent repeatability, comparable with that of SD-OCT.[11] Yang et al. used SS-OCT wide scan to measure RNFL thickness in a wide area (12 X 9 mm), and standard disc scan of SS-OCT and SD-OCT to measure cpRNFL thickness. They compared diagnostic abilities of these scan modes for glaucoma detection and reported that RNFL thickness of the wide area and circumpapillary area as measured by SS-OCT had similar diagnostic accuracy to that of cpRNFL thickness measurements obtained by SD-OCT.[10] In another study, the same team used SS-OCT wide and SD-OCT standard macula scans to measure mGCC and mGCIPL thickness, and standard disc scans of SS-OCT and SD-OCT to measure cpRNFL thickness. They evaluated the diagnostic ability of these scan modes to detect glaucoma (and early glaucoma), finding that the diagnostic ability of mGCC and mGCIPL thickness measurements of both SS-OCT and SD-OCT were similar to that of cpRNFL.[9] These previous studies compared measurements of SS-OCT with those of the corresponding mode of the SD-OCT, but did not compare measurements between the modes of SS-OCT. To our knowledge, ours is the first report to compare the measurements and diagnostic ability of wide scan and standard macula/disc scans of SS-OCT in patients with glaucoma.

The repeatability for each scan was excellent (ICC values between 0.991 and 0.999). The ICC values obtained for evaluating the agreement between wide and standard macula/disc scans also showed excellent agreement in all parameters (0.800–0.997). Similar reproducibility of the mGCIPL thickness parameters in SS-OCT wide scans (ICC values between 0.958 and 0.995) was reported in a previous study.[11] These results indicate that macular and cpRNFL parameters of SS-OCT wide scans showed good reproducibility and good agreement with SS-OCT standard macula/disc scan in general.

In the present study, the ability to detect glaucoma was similar between wide and standard macula/disc scans. The AUCs were greatest in total and inferior quadrant cpRNFL for both wide and standard disc scans. Similarly, previous studies reported that total and inferior quadrant cpRNFL parameters showed the largest AUCs (0.89 and 0.90, respectively) using SS-OCT standard disc scans,[10] and superior quadrant, total and inferior quadrant cpRNFL parameters (0.87, 0.83, and 0.79, respectively) as the top 3 cpRNFL parameters using SS-OCT wide scans.[9] For macular parameters, OT and OI subfields for both wide and standard macula scans showed the largest AUCs. This result is also in agreement with previous studies.[8, 9] The highest AUCs among mGCIPL parameters fell into the OT and OI subfields (0.825 and 0.847, respectively) in SS-OCT standard macula scan.[8] Similarly in SS-OCT wide scans, inferotemporal and superotemporal sectors among the 6 divided sectors of mGCC and mGCIPL harbored the largest AUCs (mGCC, 0.84 and 0.82, respectively; mGCIPL, 0.81 and 0.73, respectively) in another study.[9] As described in previous studies,[15, 16] the greater diagnostic abilities in outer subfields than inner subfields can be explained by higher susceptibility to glaucomatous damage in inferior arcuate fibers, followed by superior arcuate fibers, then papillomacular fibers.[17] In addition, the superior diagnostic abilities of temporal and inferior subfields may be due to differences in spatial distribution of superior and inferior retinal ganglion cell (RGC) axons. Axons of inferior macular region RGCs project to the inferior pole of the optic nerve head (ONH), while axons of the superior macular region RGCs project to the temporal ONH,[18] which is generally not involved until end stages of disease.[17] Thus, glaucomatous damage at the inferior pole of the ONH may reflect damage to the inferior and inferotemporal macular area, meanwhile glaucomatous damage at the superior pole of the ONH may not be detected in the macular analysis.

The results of this study showed generally good agreement between wide and standard macula/disc scans. However, there were tendencies in total cpRNFL, mGCIPL, and mGCC thicknesses that the difference (wide–standard macula/disc) was larger as the measurement value became thicker (Fig 3). Considering these points, it is difficult to say that they are interchangeable between the wide scan and standard macula/disc scan. Nevertheless, as wide scans show comparable diagnostic ability to the standard macula/disc scan for assessing glaucoma, the results of this study suggest that a single wide scan can replace the separate standard macula/disc scan used for evaluating glaucoma patients. Acquiring macular and circumpapillary measurements using a single wide scan is less time consuming, and minimizes alignment errors compared to using two separate scans. Patient fatigue can be reduced compared to separate scans, which can also reduce measurement errors such as fixation error, and will also lessen interference from blinking eyes while performing scans. Furthermore, SS-OCT has the advantage of higher scan speed than SD-OCT, which further improves the patient convenience and minimizes measurement errors. In addition to patient convenience, performing a single wide scan instead of a single standard macula or disc scan during routine exams in glaucoma and retina clinics can reduce the risk of missing disease, as relatively high rates of retinal comorbidity in glaucoma patients have been known.[19] In Topcon SS-OCT, wide and standard macula/disc scans use the same resolution of 512 A-scans X 256 B-scans and cover different size areas (12 X 9 mm in wide scan, 6 X 6 mm in standard macula/disc scan). Among the wide scan mode types offered by Topcon SS-OCT, 3-Dimensional 5-Line Cross mode (3D 5LC mode) provides central 5 line scans (also 12 X 9 mm) horizontally and vertically in addition to the 3-Dimensional volume scan, which provides sufficient information about the macular and circumpapillary areas in a single examination. Another strength of wide scan is that defects in 11 and 12 o’clock cpRNFL (in the right eye orientation) that appear less apparently in the macular area of standard macula scan than 6–10 o’clock cpRNFL defects[20] can be easily missed in standard macula scan, while they are hard to be missed in a single-page report of wide scan including both cpRNFL and mGCC. In addition, the RNFL thickness map included in the report of wide scan (Fig 1A) has another strength. A recent study have shown that RNFL thickness map of wider area (12 X 9 mm) provided in wide scan of SS-OCT could detect early structural changes that could not be detected well using cpRNFL or mGCIPL thickness maps and RNFL defects apart from the optic disc can be more easily visualized with the wide-field RNFL maps.[21]

There are several limitations of this study. First, the results of this study were obtained entirely from subjects of East Asian descent. Additional studies using subjects from different populations are required to generalize our conclusions. Second, as this was a retrospective chart review study, we were unable to evaluate ability to identify glaucomatous progression in each scan mode; further longitudinal studies are needed to address this question. Third, the glaucoma patients included in this study showed average MD of -5.26, which indicates that most were experiencing early to moderate glaucoma. As thickness measurements vary according to the severity of glaucoma, there may be differences in results depending on the distribution of glaucoma severity. Additionally, the distribution of IOP suggests that most of the glaucoma patients included had normal tension glaucoma; there may be differences in measurements associated with the classification of glaucoma. These questions remain to be addressed with larger studies, while considering the distribution of glaucoma. Lastly, we used only the Topcon SS-OCT among several SS-OCT models in this study. As other latest commercial models of SS-OCT also provide wide scan mode, the advantages of a single wide scan could also be applied to other models of SS-OCT.

In conclusion, the agreement of macular thickness measurements between SS-OCT wide scan and SS-OCT standard macula scan, as well as the agreement of cpRNFL measurements between SS-OCT wide scan and SS-OCT standard disc scan were both generally excellent, and the glaucoma-discriminating ability of SS-OCT wide scan was comparable to that of SS-OCT standard macular scan for macular thickness measurement and SS-OCT standard disc scan for cpRNFL thickness measurement. Considering accuracy, patient convenience, and efficiency of medical care, a single wide scan can replace separate standard macula/ disc scans when evaluating glaucoma.

## References

[1] Medeiros FA, Zangwill LM, Bowd C, Mansouri K, Weinreb RN. The Structure and Function Relationship in Glaucoma: Implications for Detection of Progression and Measurement of Rates of ChangeStructure and Function Relationship in Glaucoma. Invest Ophthalmol Vis Sci. 2012;53(11):6939–6946. doi: 10.1167/iovs.12-10345 22893677 10.1167/iovs.12-10345 PMC3466074

[2] Weinreb RN, Aung T, Medeiros FA. The pathophysiology and treatment of glaucoma: a review. JAMA. 2014;311(18):1901–1911. doi: 10.1001/jama.2014.3192 24825645 10.1001/jama.2014.3192 PMC4523637

[3] Bussel II, Wollstein G, Schuman JS. OCT for glaucoma diagnosis, screening and detection of glaucoma progression. Br J Ophthalmol. 2013:bjophthalmol-2013-304326. 10.1136/bjophthalmol-2013-304326 PMC4208340 24357497

[4] Leung CK-S. Diagnosing glaucoma progression with optical coherence tomography. Curr Opin Ophthalmol. 2014;25(2):104–111. doi: 10.1097/ICU.0000000000000024 24370973 10.1097/ICU.0000000000000024

[5] Hood DC, Raza AS, de Moraes CGV, Liebmann JM, Ritch R. Glaucomatous damage of the macula. Prog Retin Eye Res. 2013;32:1–21. doi: 10.1016/j.preteyeres.2012.08.003 22995953 10.1016/j.preteyeres.2012.08.003 PMC3529818

[6] Mansouri K, Medeiros FA, Marchase N, Tatham AJ, Auerbach D, Weinreb RN. Assessment of choroidal thickness and volume during the water drinking test by swept-source optical coherence tomography. Ophthalmology. 2013;120(12):2508–2516. doi: 10.1016/j.ophtha.2013.07.040 24021895 10.1016/j.ophtha.2013.07.040 PMC3833954

[7] Hirata M, Tsujikawa A, Matsumoto A, Hangai M, Ooto S, Yamashiro K, et al Macular choroidal thickness and volume in normal subjects measured by swept-source optical coherence tomography. Invest Ophthalmol Vis Sci. 2011;52(8):4971–4978. doi: 10.1167/iovs.11-7729 21622704 10.1167/iovs.11-7729

[8] Lee KM, Lee EJ, Kim TW, Kim H. Comparison of the Abilities of SD-OCT and SS-OCT in Evaluating the Thickness of the Macular Inner Retinal Layer for Glaucoma Diagnosis. PLoS One. 2016;11(1):e0147964 doi: 10.1371/journal.pone.0147964 26812064 10.1371/journal.pone.0147964 PMC4727815

[9] Yang Z, Tatham AJ, Weinreb RN, Medeiros FA, Liu T, Zangwill LM. Diagnostic ability of macular ganglion cell inner plexiform layer measurements in glaucoma using swept source and spectral domain optical coherence tomography. PLoS One. 2015;10(5):e0125957 doi: 10.1371/journal.pone.0125957 25978420 10.1371/journal.pone.0125957 PMC4433247

[10] Yang Z, Tatham AJ, Zangwill LM, Weinreb RN, Zhang C, Medeiros FA. Diagnostic ability of retinal nerve fiber layer imaging by swept-source optical coherence tomography in glaucoma. Am J Ophthalmol. 2015;159(1):193–201. doi: 10.1016/j.ajo.2014.10.019 25448991 10.1016/j.ajo.2014.10.019 PMC4293127

[11] Lee SY, Bae HW, Kwon HJ, Seong GJ, Kim CY. Repeatability and Agreement of Swept Source and Spectral Domain Optical Coherence Tomography Evaluations of Thickness Sectors in Normal Eyes. J Glaucoma. 2017;26(2):e46–e53. 27599180 10.1097/IJG.0000000000000536

[12] Alshareef RA, Dumpala S, Rapole S, Januwada M, Goud A, Peguda HK, et al Prevalence and distribution of segmentation errors in macular ganglion cell analysis of healthy eyes using Cirrus HD-OCT. PLoS One. 2016;11(5):e0155319 doi: 10.1371/journal.pone.0155319 27191396 10.1371/journal.pone.0155319 PMC4871429

[13] Cicchetti DV. Guidelines, criteria, and rules of thumb for evaluating normed and standardized assessment instruments in psychology. Psychol Assess. 1994;6(4):284.

[14] Morooka S, Hangai M, Nukada M, Nakano N, Takayama K, Kimura Y, et al Wide 3-dimensional macular ganglion cell complex imaging with spectral-domain optical coherence tomography in glaucoma. Invest Ophthalmol Vis Sci. 2012;53(8):4805–4812. doi: 10.1167/iovs.12-9870 22695956 10.1167/iovs.12-9870

[15] Nakano N, Hangai M, Nakanishi H, Mori S, Nukada M, Kotera Y, et al Macular ganglion cell layer imaging in preperimetric glaucoma with speckle noise–reduced spectral domain optical coherence tomography. Ophthalmology. 2011;118(12):2414–2426. doi: 10.1016/j.ophtha.2011.06.015 21924499 10.1016/j.ophtha.2011.06.015

[16] Nakatani Y, Higashide T, Ohkubo S, Takeda H, Sugiyama K. Evaluation of macular thickness and peripapillary retinal nerve fiber layer thickness for detection of early glaucoma using spectral domain optical coherence tomography. J Glaucoma. 2011;20(4):252–259. doi: 10.1097/IJG.0b013e3181e079ed 20520570 10.1097/IJG.0b013e3181e079ed

[17] Quigley HA, Addicks EM. Regional differences in the structure of the lamina cribrosa and their relation to glaucomatous optic nerve damage. Arch Ophthalmol. 1981;99(1):137–143. 7458737 10.1001/archopht.1981.03930010139020

[18] Bagga H, Greenfield DS, Knighton RW. Macular symmetry testing for glaucoma detection. J Glaucoma. 2005;14(5):358–363. 16148583 10.1097/01.ijg.0000176930.21853.04

[19] Griffith JF, Goldberg JL. Prevalence of comorbid retinal disease in patients with glaucoma at an academic medical center. Clin Ophthalmol. 2015;9:1275–1284. doi: 10.2147/OPTH.S85851 26203217 10.2147/OPTH.S85851 PMC4508087

[20] Kim KE, Park KH, Yoo BW, Jeoung JW, Kim DM, Kim HC. Topographic localization of macular retinal ganglion cell loss associated with localized peripapillary retinal nerve fiber layer defect. Invest Ophthalmol Vis Sci. 2014;55(6):3501–3508. doi: 10.1167/iovs.14-13925 24801510 10.1167/iovs.14-13925

[21] Lee WJ, Na KI, Kim YK, Jeoung JW, Park KH. Diagnostic Ability of Wide-field Retinal Nerve Fiber Layer Maps Using Swept-Source Optical Coherence Tomography for Detection of Preperimetric and Early Perimetric Glaucoma. J Glaucoma. 2017;26(6):577–585. doi: 10.1097/IJG.0000000000000662 28368998 10.1097/IJG.0000000000000662

